# On the Moral Pathology of London

**Published:** 1858-10-01

**Authors:** 


### Art. II.—ON THE MORAL PATHOLOGY OF LONDON.

The emotion which Dante underwent when, in a vision, he was suddenly removed from earth and placed within the confines of hell, was not unlike that which would be experienced by one who, for the first time, was transported from a main thoroughfare of London into the midst of one of those dense masses of building in which the nether class of the metropolitan population mainly dwells, and in which utter poverty, squalid wretchedness, and flagrant villany have their home.

As the boundaries of the metropolis have extended, certain results have come to pass, which are in some respects similar to those which may be traced as having happened during the growth of several of the great cities of India. In them, as successive dynasties have widened the area of a city, the older portions have been deserted and suffered to fall into ruin, and ultimately they have become the haunt of the jackal and the hooded snake; and while London has been growing vaster and vaster, and its streets have been spreading out still more widely, the wealthier classes of the metropolis have gradually deserted the older and closer portions of the city. These, forgotten altogether by the many, have fallen through various stages of degradation, and in the end the dilapidated, and even ruinous buildings have become the sad homes of those who, from poverty or from vice, have wished to hide themselves from the common eye, and who have found a secure retreat in the midst of the brick-and-mortar jungles of the metropolis.

Narrow and little-known streets traverse these districts, and their cores are pierced with courts which reek with filth, and which are contracted and irregular as if they had been painfully gnawn out of the huge blocks of brickwork. The houses are foul and dilapidated without, but they are fouler and more ruinous within. The walls are stained with damp, and the floors are rickety. The wood-work is black with age and filth, and it swarms with vermin. The ceilings are broken and discoloured. The windows are shattered, and paper and rags take the place of glass. Every room in these houses shelters a separate family, and every house is tainted throughout with foul odours, which steam from the open doorway and raised windows into the court-yards and streets. The door-steps are littered with children whose contracted and impish features parody a maturer age; and men and women, whose faces are graved deeply with those lines which tell of that terrible fight for a livelihood in which, as one of their own class said, "They don't find a living, it's only another way of starving," or whose aspect is that of bold, open, and perhaps full-fed villany, lounge upon the pathway.

Cradled in such scenes a child is not merely familiarized, it is naturalized, with wretchedness, filth, and vice. It receives little parental care, for the parents are too commonly engaged in those fierce, never-faltering efforts by which alone they can scrape together the barest means of subsistence. The mother may not, if she would, give much attention to her offspring, and too often, alas! the incessant occupation of the mind in that toil by which the food necessary to sustain human life has to be obtained, and the perverted and deadened feelings which arise from the continued struggle with want, blunt the mother's affection towards her child, and it receives from her only those cares which arise from a savage necessity. Coarsely, often insufficiently, and usually irregularly fed, the little being learns at the very threshold of life to endure with more or less patience the cravings of hunger; its intellect is first stirred into action in seeking the means to satisfy its most urgent physical wants; and often it exhibits a painful precocity in those matters which are needful to procure a mere subsistence:—

"'I go about the streets with water-creases,'" said a little girl, who, "although only eight years of age," writes Mr. Mayhew, "has entirely lost all childish ways, and was indeed in thoughts and manner a woman. There was something cruelly pathetic in hearing this infant, so young that her features had scarcely formed themselves, talking of the bitterest struggles of life with the calm earnestness of one who had endured them all. I did not know how to talk to her."—"'I go about the streets with water-creases, crying four bunches a penny, water-creases,'" said this girl. "'I am just eight years old—that's all; and I've a big sister and a brother, and a sister younger than I am. On and off, I've been very near a twelvemonths in the streets. Before that I had to take care of a baby for my aunt. No, it wasn't heavy—it was only two months old; but I minded it for such a time till it came to walk. It was a very nice little baby, not a very pretty one, but if I touched it under the chin it would laugh. Before I had the baby I used to help mother, who was in the fur trade, and if there was any slits in the fur I'd sow them up. My mother learned me to needlework and to knit when I was about five. . . . . I have to be down at Farringdon-market between four and five, or else I can't get any creases, because every one almost—especially the Irish—is selling them, and they're picked up so quick. . . . . When we've bought a lot, we sits down on a door-step and ties up the bunches. We never goes home to breakfast till we've sold out; but if it's very late, then I buys a pen-north of pudden, which is very nice with gravy. I don't know hardly one of the people as goes to Farringdon, to talk to; they never speaks to me, so I don't speak to them. We children never play down there, 'cos we're thinking of our living. . . . . It's very cold before winter comes on reg'lar—'specially getting up of a morning. I gets up in the dark by the light of the lamp in the court. When the snow is on the ground, there's no creases. I bears the cold—you must; so puts my hands under my shawl, though it hurts 'em to take hold of the creases, especially when we takes 'em to the pump to wash 'em. No; I never see any children crying—it's no use. . . . . I don't have no dinner. Mother gives me two slices of bread and butter and a cup of tea for breakfast, and then I go till tea, and has the same. We has meat of a Sunday, and, of course, I should like to have it every day. Mother has just the same to eat as we has, but she takes more tea—three cups sometimes. . . . . I am a capital hand at bargaining—but only at buying water-creases. They can't take me in. If the woman tries to give me a small handful of creases, I says, I ain't a goin' to have that for a ha'porth, and I go to the next basket, and so on all round. I know the quantities very well. For a penny I ought to have a full market hand, or as much as I could carry in my arms at one time without spilling. For threepence I has a lap full, enough to earn about a shilling; and for sixpence I gets as many as crams my basket. I can't read or write, but I knows how many pennies goes to a shilling, why, twelve, of course, but I don't know how many ha'pence there is, though there's two to a penny. When I have bought threepen-north of creases, I ties 'em up into as many little bundles as I can. They must look biggish, or the people wont buy them; some puffs them out as much as they'll go. All my money I earns I puts in a club, and draws it out to buy clothes with. It's better than spendin' it in sweet stuffs for them as has a living to earn. Besides, it's like a child to care for sugar-sticks, and not like one who's got a living and vittals to earn. I ain't a child, and I sha'n't be a woman till I'm twenty, but I'm past eight, I am. I don't know nothing about what I earns during the year, I only knows how many pennies goes to a shilling, and two ha'pence goes to a penny, and four fardens goes to a penny. I knows, too, how many fardens goes to tuppence—eight. That's as much as I wants to know for the markets.' "*

The first smatterings of a child are commonly in a language which is very peculiar. It teems with words which are in use among the nether class alone. Remnants of the gipsy tongue may perhaps form a part of this language, but in the main it consists of words which, seemingly, have been originally invented, with strange ingenuity, for the readier concealment of the peculiar habits of the different segments of the nether class, and it is thoroughly fitted for that purpose, and also to express the most depraved passions of man. With this language the child is familiarized the moment he begins to frequent the streets, if not from the cradle; and long before the meaning of the words uttered can be fully comprehended, a child will become practised in the use of the most repulsive phrases of the nether tongue. The shrill oaths and the foul expressions which may be heard bandied about among the children of the nether class are used as common forms of speech, of the actual signification of which the child is generally ignorant.

The crowded room which forms the child's home, and in which the whole family herd together night and day, and the whole of the domestic arrangements have to be carried out, gives rise to habits which are destructive of all the ordinary feelings of modesty and decency; and in the streets the sensual passions are early aroused to full swing by the constant association of the two sexes. The passer-by may witness, in the by-paths and close alleys,

* "London Labour and London Poor," by Henry Mayhew, vol. i. pp. 151–2.

among the children which tumble about and roll upon the pathway (in their degradation not unlike the wild dogs which are found in the streets of a Turkish town), the first stages of that excessive sensual depravity which characterizes the maturer years of the nether class. Moreover, the wild, untrained life in which these children grow during their earlier years, unfits them almost ever after for the steady and persistent labour of a handicraftsman. It is certain that they will have to struggle sternly for their bread, and that, too, from early morn till night, day by day, but they will have recourse solely to such labour as will give them the freedom of moving about as they list, and which will keep them in the open air, and near the constant turmoil and excessive excitement of a large town.

So soon as the child becomes strong enough to aid in getting its own living, it is at once set to work in one of those occupations which alone are open to it. It is taught the necessity of perfect honesty to its own class, and those sleights of dealing by which the profits of the occupation it is learning may be increased. Already grounded in the language and actions of vice, the child enters into more intimate association with those who have already matriculated in the cares, the vices, and the pleasures of the streets, and listening with greediness to the vicious achievements of older children, it seeks to emulate their actions. The lad of eight years of age may be seen copying the matured bestiality of his elders, gambling with the few halfpence he may chance to have picked up, and aping the man at the bar of the dram-shop; while the childish girl will often shock the ears and offend the eyes by her filthy language and still filthier actions—nay, the teens of years will barely have been reached, before both boys and girls will have become fully trained in everything that is vile and vicious.

The full-grown lad is characterized usually by great quickness in whatever relates to his peculiar mode of life. He rarely misses the opportunity of "turning a penny;" and he is thoroughly initiated in all the wiles of his irregular existence. Of virtue he knows nothing: the burglar and highwayman are his chief heroes, and he looks upon the successful thief with admiration. He has but a vague notion of the wrongfulness of thieving; his idea of a thief, indeed, is somewhat similar to that entertained by the ordinary run of people about a soldier. Many admire and envy the successful soldier, but comparatively few will undergo the danger necessary to acquire the envied distinction; and the lad of the nether class envies the boldness of the thief, but he does not care to undergo the peril of thieving; but he hates the police with a perfect hatred, and he regards the law either as a necessary evil to which his class is exposed, or as an engine put in force for the special benefit of the higher classes. He is singularly ignorant of most things beyond the requirements of a street-life, and this ignorance often contrasts strongly with the sharpness and cunning which he displays in procuring his livelihood.

"Yes," said a street-lad, of thirteen or fourteen years of age, to Mr. Mayhew, "he had heer'd of God who made the world. Couldn't exactly recollec' when he'd heer'd on him, but he had, most sarten-ly. Didn't know when the world was made, or how anybody could do it. It must have taken a long time. It was afore his time, or 'yourn either, sir.' Knew there was a book called the Bible; didn't know what it was about; didn't mind to know; knew of such a book to a sartinty, because a young 'oman took one to pop [pawn] for an old 'oman who was on the spree—a bran new 'un—but the cove wouldn't have it, and the old 'oman said he might be d——d. Never heer'd tell on the deluge; of the world having been drownded; it couldn't, for there wasn't water enough to do it. He weren't agoing to fret hisself for such things as that. Didn't know what happened to people after death, only that they was buried. Had seen a dead body laid out; was a little afeard at first; poor Dick looked so different, and when you touched his face, he was so cold! oh, so cold! Had heer'd on another world; wouldn't mind if he was there hisself, if he could do better, for things was often queer here. Had heer'd on it from a tailor—such a clever cove, a stunner—as went to 'Stralia [Australia], and heer'd him say he was going into another world. Had never heer'd of France, but had heer'd of Frenchmen; there wasn't half a quarter so many of 'em as of Italians, with their earrings like flash gals. Didn't dislike foreigners, for he never saw none. What was they? Had heer'd of Ireland. Didn't know where it was, but it couldn't be very far, or such lots wouldn't come from there to London. Should say they walked it—ay, every bit of the way, for he'd seen them come in all covered with dust. Had heer'd of people going to sea, and had seen the ships in the river, but didn't know nothing about it, for he was very seldom that way. The sun was made of fire, or it wouldn't make you feel so warm. The stars was fire, too, or they wouldn't shine. They didn't make it warm, they was too small. Didn't know any use they was of. Didn't know how far they was off; a jolly lot higher than the gas-lights some on 'em was. Was never in a church; had heer'd they worshipped God there; didn't know how it was done; had heer'd singing and playing inside when he'd passed; never was there, for he hadn't no togs to go in, and wouldn't be let in among such swells as he had seen coming out. Was a ignorant chap, for he'd never been to school, but was up to many a move, and didn't do bad. Mother said he would make his fortin yet. Had heer'd of the Duke of Wellington; he was old Nosey; didn't think he ever seed him, but had seed his statty. Hadn't heer'd of the battle of Waterloo, nor who it was atween; once lived in Webber-row, Waterloo-road. Thought he had heer'd speak of Buonaparte; didn't know what he was; thought he had heer'd of Shakespeare, but didn't know whether he was alive or dead, and didn't care. A man with something like that name kept a dolly, and did stunning; but he was such a hard cove that if *he* was dead it wouldn't matter. Had seen the Queen, but didn't recollec' her name just at that minute; oh! yes, Wictoria and Albert. Had no notion what the Queen had to do. Should think she hadn't such power (he had first to ask me what 'power' was) as the Lord Mayor, or as Mr. Norton as was the Lambeth beak, and perhaps is still. Was never once before a beak, and didn't want to. Hated the crushers [police]; what business had they to interfere with him if he was only resting his basket in a street? Had been once to the Wick [Victoria Theatre], and once to the Bower: liked tumbling better; he meant to have a little pleasure when the peas came in."*

* "London Labour and London Poor," vol. i. p. 474.

The licentiousness and depravity of both boys and girls of the nether class are astounding. It is not uncommon for a lad of fourteen years of age, if he has succeeded in obtaining a livelihood, to form a companionship with a girl, and both leaving home they will live together as man and wife. No bond but sensual fancy binds the couple together, and friendly exchanges of "partners" are not unknown between lads. Notwithstanding this laxity of affection, however, the lads are usually fiercely jealous, and they will often treat their girls with great brutality, on the slightest provocation; on the other hand, the girls will freely prostitute themselves, in order to gratify a want of the boy with whom they are united. Many girls have lost their chastity long before they know the signification of the term; and by the nether class marriage is regarded with the utmost indifference, and it is commonly looked upon as a most unnecessary rite. One of the principal results of the close packing of families, male and female, in one room, and of the low lodging-houses where men and women herd together in the same apartment, is to pervert or destroy all notions of modesty and virtue, and to degrade the passions of both children and up-grown persons to the level of those of the dog. The horrible scenes of immorality enacted in the low lodging-houses, which are the great resort of lads living in companionship with girls, of vagrants, and of thieves, are indescribable. The surveillance of the police and the requirements of the law are doing much, however, to restrain the vice of these houses within less rampant bounds.

Personal indifference to pain is a common matter of boast among the boys, and they manifest little sympathy for the sufferings of others. Both boys and girls are loud in anger, and the latter admire pugilism, but they are not much addicted to fighting among themselves. The boys delight in dog-fighting and rat-catching, but their chief amusements are gambling (which is usually an extravagant passion with them, and one very early developed) and the cheap theatres. A certain degree of vanity in dress is occasionally manifested, and will at times incite a lad to unusual exertions to gratify his taste; and not unfrequently, if the pocket will permit, and the boy's gains have not been swallowed up by gambling, the palate will be indulged to excess in sweetmeats and rich food, and perhaps also in spirituous liquors.

The same traits that characterize the boy and girl, stamp also the full-grown man and woman: vagrant in habits—ignorant of all beyond the readiest modes of obtaining a livelihood—haters of the law—admirers of successful crime—knowing little or nothing, or indifferent to morality and virtue—sensual even to the terrible climax of incest—eminently brutal—utterly improvident, indeed, ignorant of the value of saving, when there may be an opportunity of laying money by—at one time pandering to the appetite by spirits and food, at another bearing semi-starvation with singular stolidity—and not only ignorant of the higher duties of life, but wanting the preliminary knowledge by means of which those duties may be comprehended.

"Visiting a sick man," writes Mr. Vanderkiste, "with one new missionary, I requested him to read and instruct him, which he did, detailing to him our fallen condition, our need of salvation, and the redemption purchased for us, in a very correct manner, and then reading a portion of a chapter in the Gospels in proof of what he had said. The poor man listened with every appearance of attention, and when my young friend said, 'You know Mr. ——,' or any other interrogatives, he replied, 'Certainly, sir;' or, 'In course, sir.' My companion appeared pleased with the man's attention to instruction, and I thought it time to undeceive him. 'Mr. ——,' said I, 'my friend has been taking much pains to instruct you, and now I will ask you a few questions. Do you know who Jesus Christ was?' 'Well, no,' said he, after a pause, 'I should say that's werry hard to tell.' 'Do you know whether he was St. John's brother?' 'No, that I don't.' 'Can you tell me who the Trinity are?' 'No, sir.' 'Are you a sinner?' 'Oh, certainly, sir, we are all sinners.' A pause. 'Have you ever done wrong?' 'Why, no, I don't consider as ever I have.' 'Did you ever commit sin?' 'Why, no, I don't know as ever I did.' 'But do you think you're a sinner?' 'Oh, certainly, sir, we're all sinners' 'What is a sinner?' 'Well, I'm *blest* if I know rightly; I never had no head-piece.'"*

Dark ignorance, and an entire absence of moral feeling, are the grand characteristics of the nether class; and they are the natural results of the circumstances under which that class exists. The foul and fetid dwellings, the herding together of families as if they were wild beasts, and the entire swallowing up of the mind in the struggle for daily bread, are main steps in the sad genesis of that degradation, both physical and mental, which is witnessed among this ill-starred people. It is difficult, perhaps impossible, to estimate the number of the nether class of the metropolitan population; but it forms a mass which exerts a too manifest influence upon the social condition of the metropolis. Having no fixed political knowledge, but hating blindly all that is placed above it by comfort or wealth; ignorant of the meaning and end of the law, and detesting its officers; and living in habits and indulging ideas of life which are altogether subversive of social stability, this class forms an unstable mass, which is ever ripe for the worst form of demagogue to work upon: and still worse, the ordinary habits of the individuals who form the nether class are separated in so slight a degree from that of actual crime, that every change in the condition of the class (whether it be for the better, and thus furnish the means for gratifying the inor-

* "Notes and Narratives of a Six Years' Mission, principally among the Dens of London. By R. W. Vanderkiste, late London City Missionary." Third Edition, p. 37.

dinate desire for sensual pleasures, or for the worse, and causes suffering and want) is apt to destroy, in many instances, the slight barrier that separates the common feelings and practices of the class from overt criminality; and thus the nether class becomes a source from which is constantly flowing a greater or less stream of crime.

In "good times" the goodness is measured by the amount of gain which may be devoted to the dram-shop, to gambling, and to profligacy, and both men and women every moment float into crime on our modern Phlegethon, the river of "strong drink" (for once in the deep stream, one may not hope to escape from the current scathless, banked in as it is by profligacy and ruin), while the fierce excitement of gambling continually pricks the loser beyond the pale of the law; and in "bad times" the feeling grows apace which was aptly expressed by a ticket-of-leave man in 1857: "I don't see why I should starve; and I'm not going to do it." Action is only wanted to convert such a thought into open crime.

Again, the brutality of a father, the harshness of a mother, the sufferings of a week's hunger, the unsettledness of a vagrant life, the temptations arising from a false code of morality, the passion for sensual and other pleasures, all pave a broad and easy pathway, along which the children of the nether class troop to crime, and, if need be, a guide and tempter is never wanting in the practised criminal, who is always at hand.

It may be wondered how, with so unstable a class of the population living in our midst, suffering so much, and yet so little bound by the laws of morality, we should be for one moment safe in our houses, and that crime and villany should not be more rampant than they are. But there is never wanting among the nether class a vague but salutary notion—one which the individuals of the class would themselves find difficult to define, and which, indeed, has more the character of a superstition than anything else—of the supreme power of the law, and of the higher classes of society; and crime is almost always regarded as a final and irremediable step. Moreover, a slight moral influence—"a little glooming light, much like a shade"—(which is not to be lost sight of in estimating the restraining powers at work among the nether class) is brought to bear upon the class by the intermixture with it of many who have had the advantage of a moral training, and who from misfortune have had to seek a home in the sinks of the metropolis, but whose moral faculties have not been obliterated.

The nether class of the metropolitan population is not recruited within itself alone. It receives constant accessions from those who, being reduced to extreme poverty, are compelled to seek a refuge in the worst haunts of the metropolis, and to pick up a living in the streets; it is the abyss into which fall those ill-fated children who flee from their homes on account of cruel usage, who are outcast from desertion or from being orphans, or who, from a perverted love of a *quasi*-idle and wandering life, prefer the wild and irregular mode of existence of a street lad. The nether class is also the great receptacle into which fall those who, degraded by flagrant vice, sink step by step from a higher grade of society, until they at length plunge into this deep slough of humanity. Those who have been driven by poverty and want into the haunts of the nether class, have among them many who have not wanted a greater or less degree of education and moral tuition, and who, notwithstanding the deadening effects of their impoverished state, still retain much of their better nature, and by their moral influence exert some slight check upon their neighbours; but those who have been degraded by their ill-regulated passions, commonly outgo, in the foulness of their vice, that with which they are brought into contact. They exercise their more fully-developed mental faculties to give a higher finish to villany; they study to give language its highest power for the suggestion and fulfilment of immorality; they become the arch-teachers of devilism; they, in fact, show but too fully that the most thorough villain is the best-educated villain.

But there is a section of the nether class—the street Irish—which, although found in the same haunts, and exposed to the same struggles for existence, and to the same temptations and vice, differs greatly in the character of its morality from the ordinary members of the class. In the heart of London, the emigrant Irish preserve almost intact the peculiar traits which distinguish them in their own country. They retain in a great measure their prejudices against the English, their warm attachment to their own family, their habits of herding together and of feeding on the coarsest food, their excitable passions (which often lead them to break the law), and their ready wit and tongue. They also maintain, in a great measure, their aversion to steady and protracted labour, and without shame, or with indifference, have recourse to beggary or the workhouse. The majority of the street Irish are Roman Catholics, and they keep their hold of Romanism and that blind faith in the priest which is only found—at least, in this country—among the most ignorant professors of that religion. But the chief moral characteristic of the street Irish, as compared with the remainder of the nether class, is their freedom from wantonness. The females retain their virtue in the deepest sinks of vice; and the testimony is general, that when the Irish females do fall into immoral courses, it is from the unfavourable influences to which they are exposed by constant association with vice.

The promiscuous herding together of males and females in the same apartment at night, and the intimate association of the young of both sexes, are not necessary causes of immorality. It is only when these causes co-exist with loose or imperfect notions of morality, that we find the sad results which are too commonly witnessed among the nether class of the English in large towns. Among the Romanist Irish, a stern notion of morality is invariably found, partly traditional, mainly, perhaps, a result of religious teaching; for it matters not how destitute and impoverished an Irish Romanist may be, it is rare for him to be beyond the influence of religious teaching, and the doctrines of morality, which are taught side by side with his religious duties, are also practised with as great regularity. Moreover, the Irishman, even in England, is not often exempt from the supervision of the priest, who has an individual influence over his flock of vast power in teaching and restraining.

A criminal section of the nether class is one of the necessary results of the physical and moral circumstances under which the class exists. But the nucleus of the criminal section is not formed of individuals who have had recourse to crime incidentally, or who have been driven to crime by necessity, but mainly of persons who are of criminal parentage, and who have been specifically trained to crime, and to whom crime is simply the business of life. Hereditary crime cannot, however, often boast of such long descent that its origin may not be readily traced. The course of the law usually cuts short the development of family crime before it has attained to the growth of a third or fourth generation. The common criminal possesses in excess all the evil qualities of the class from which he is an offshoot; and the low cunning which he shows in his pursuits is no greater in degree, although it may be much more striking in appearance, than that which is displayed by the nether class in more honest endeavour to obtain a livelihood. The ill-concealed feeling of admiration which is entertained by a large portion of the nether class for individuals who have dared to break the laws; the excitement consequent upon the act of crime itself; the apparently easy mode in which, by pursuing criminal courses, money may be obtained, comparative idleness indulged in, and a passion for gambling or other pleasures gratified, have a peculiar fascination for many young criminals. "Lord, how I do love thieving!" exclaimed a young thief in Colonel Chesterton's hearing, "if I had thousands, I'd still be a thief."*

By the thoroughly trained thief the law is regarded much in the same way as ordinary individuals regard sickness. The evil

* "Revelations of Prison Life, with an Inquiry into Prison Discipline and Secondary Punishments," by Geo. Laval Chesterton. 1856. Vol. i. p. 165.

is a necessary one, and it is to be avoided, if practicable; but should a casualty befal, it must serve as a lesson by which, in future, mischief may be more readily shunned. An efficient police has done much to diminish the halo which hangs about crime and the criminal in the haunts of the nether class, but still the false lustre lingers and serves as a stimulant to the juvenile criminal.

Although the nether class of the London population has its peculiar haunts, and its depravity and licentiousness are, in a great measure, kept within itself and hid from the common eye, except in so far as they are made known by overt crime, or, perhaps, by a popular commotion, it would be a serious error to suppose that the social influence of this class does not extend beyond its capacity to nurture crime and to furnish a portion of the material by which popular tumults may be carried out. The nether class has more or less intimate bonds with the operative class on the one hand, and with the commercial class, through various kinds of tradesmen, on the other, and a baneful effect is produced upon the morality of both classes by the influence of the nether class. Moreover, the baneful effect of this influence is probably felt in every grade of society, however remote it may be in appearance from the exercise of such an influence. Certainly in the metropolis the nether class is more separated from the moral restraints of other classes than in smaller towns, and in this greater degree of separation consists one of the principal characteristics of the metropolis. In small towns the worst districts of the town are usually well open to the eye of the classes having a higher social grade than the nether class, and an indirect moral influence is brought to bear upon that class which is not without benefit. Moreover, in small towns the worst districts are easier of access to ministers of the Gospel. In the metropolis, however, where wide areas are inhabited by the nether class alone, and where these districts are almost unknown but to this class and to the policeman, an indirect moral influence of the kind mentioned is but slightly felt; but, on the other hand, it is probable that the greater amount of intense vice (not greater intensity of vice, for the difference is most probably in quantity not quality) in these districts acts much more sensibly upon the classes thrown into contact with the nether class than elsewhere.

The operative class is thrown into intimate connexion, both in business and in pleasure (particularly in those places of common resort, the dram-shop and beer-house) with that portion of the nether class which is known as the "street-folk," and it becomes to a greater or less extent familiarized with the laxity, or rather the absence of moral and religious feeling among the latter. This familiarity with the aspect and form of vice has a most injurious but insidious influence upon the moral faculties, perverting their manifestations and blunting their sensibility. Children are very susceptible of these evil effects, and the children of the operative class have not, neither can they have, as a rule, that constant maternal care which is essential to the thorough well-being of a child. The necessity which very commonly exists for every able member of an operative's family to aid in the maintenance of the family, renders it requisite that the child should be left usually in the charge of a younger member of the family, or of some person who does not possess that full interest in the welfare of the child which the mother alone feels. The child is thus left during a great portion of the day to seek its own amusement, almost uncared for, among its fellows in the streets and alleys, and there it has constantly thrown in its way waifs of the foul language, and hints of the foul habits of the "street-children," which it too readily adopts, while the grosser passions of the child, in great measure uncontrolled, are rapidly developed. Even during the school-days of children of the operative class the moral faculties are too frequently still further stunted, for the education consists often of the rudimentary forms of learning alone, the moral faculties being left altogether untilled, or if tilled, the process is of so imperfect a character as to interpose only a slight obstacle to the evil influences to which the child is exposed.

It is in childhood that the greatest mischief to morality is done. The susceptibility of the child to sensuous impressions, and the inordinate development of the passions in comparison to the intellect and moral faculties, is the chief cause of that blunted moral sensibility which is observed in after-life in almost all classes of society. The after play of the organism by means of which mental action is displayed is determined chiefly in childhood and youth, and if the passions be developed disproportionately to the intellect and moral faculties, the disproportion is commonly continued to the remotest period of life. If, indeed, the action of any portion of the material framework of thought be exaggerated or warped in youth, its effects will be manifested to a greater or less extent ever after. The influence which is exercised by the nether class upon that portion of the operative class with which it is most closely brought into contact tends to blunt the moral susceptibilities of the class, and, according to our own observation, it contributes in a marked degree towards the formation of that weakened sense of morality which is found prevalent among a large section of the operative class.

The nether class also exercises an indirect but important influence upon the commercial class. A large number of the retail dealers who supply the nether class with articles of consumption are of that class, and consequently partake of all its habits and feelings. But, in addition, the wants of the nether class are supplied by numerous tradesmen who possess education and substance, but who practise to an extraordinary extent those petty frauds of trade (petty in name only) which consist particularly in the adulteration of food and drink. The nether class suffer much from these frauds, and the inability of individuals of the class to protect themselves, their ignorance, and their desire, from poverty, to obtain cheap articles, forms a great temptation to tradesmen to practise a system of petty fraud for the augmentation of trade profits. The system of petty trade frauds is not confined to the tradesmen dealing principally with the nether class, but it is found, perhaps, in greatest intensity among them. The majority of the petty trade frauds are, perhaps, practised by the retail dealer; but many of the more serious frauds, particularly in the adulteration of articles of consumption, cannot be carried out without the co-operation of the manufacturer or wholesale dealer; hence the system of petty fraud practised by the lowest branch of a trade is found to extend its influence through every branch of the same trade. The petty frauds common among shopkeepers form a *quasi*-justification for fraud which the street dealer is quick to seize upon:—

"It ain't *we*," remarked a street tradesman, "as makes coffee out of sham chicory; it ain't *we* as makes cigars out of rhubarb leaves; *we* don't make duffers' handkerchiefs, nor weave cotton things and call them silk. If we quacks a bit, does *we* make fortins by it as shopkeepers does with their ointments and pills? If we give slang weights, how many rich shopkeepers is fined for that there? And how many's never found out? and when one on 'em's fined, why, he calculates how much he's into pocket, between what he's made by slanging, and what he's been fined, and on he goes again. *He* didn't know that there ever was short weight given in his shop; not *he!* No more do *we* at our stalls or barrows! Who 'dulterates the beer? Who makes old tea-leaves into new? Who grinds rice among pepper? And as for smuggling,—but nobody thinks there's any harm in buying smuggled things. What we does is like that pencil you're writing with to a great tree, compared to what the rich people does."*

In addition to the baneful influence to which the operative class is exposed by its more or less intimate connexion with the nether class, it is also liable to be affected by other agencies which tend to blunt or weaken the moral faculties. Of these agencies the confined dwellings of the class, and the physical requirements, if we may so speak, of relaxation hold a prominent place. One of the most powerful causes tending to blunt the morality of the operative class, is the house which has but one room, or the house which has only a single bed-chamber. When all the members of a family, parents and children, male and female, occupy the same apartment at night, it has the effect of deadening the sense of modesty and

* "London Labour and London Poor," p. 474.

decency among the children, and it leads to a weakening of the moral faculties and to an impurity of thought, the importance of which can with difficulty be appreciated to its full extent. Even the strictest religious training is barely sufficient to obviate the evil effects which may arise from this source. The sensual passions of children are very readily developed, even when the child itself knows nothing of the nature of the feelings which it experiences; but the single-chambered dwelling, or the house with one bed-chamber, occupied by several individuals of different ages and sexes, early gives a directive tendency to the sensual passions, and a proclivity to active immorality of the very gravest character. It is not, however, in the direct production of early overt immorality that the evil is most witnessed, but in a diminution of the feelings of modesty, and a proclivity to sensuality, which is shown, perhaps, in its most serious character among young females. The effects of this sensual proclivity may be readily witnessed in the lax conversation and manners which, short of actual immorality, occur in large mills or workrooms, where females of the operative classes are employed, and where no moral superintendence is exercised; but its worst effects extend into every grade of society. For, from the children of the operative class, the ranks of domestic servants are mainly recruited, and to the charge of these servants the children of the middle and higher classes of society, in infancy and early youth, are principally committed, and it frequently happens that the low grade of morality of the nurse or the housemaid either degrades or perverts the moral faculties of the most carefully trained child. The scene depicted in the "Winter's Tale" is but a faint outline of what frequently occurs in our own homes:—

"*1st Lady.* Come, my gracious lord,
Shall I be your playfellow?
*Man.* . . No, I'll none of you.
*1st Lady.* Why, my sweet lord?
*Man.* . . You'll kiss me hard; and speak to me as if
I were a baby still.
* * * * *
*2nd Lady.* Hark ye:
The queen your mother rounds apace; we shall
Present our services to a fine new prince,
One of these days; and then you'd *wanton* with us,
If we would have you."—*Act* ii. sc. 1.

The young man who begins to breast the world with a proclivity towards sensuality, derived from the circumstances of his childhood, be he poor or rich, usually leaves behind him a broad wake of sin; the young woman, to whatever class of society she may belong, is apt to be overwhelmed by the first heavings of passion or the earliest promptings of vanity. It is the blunted moral sense and the proclivity to sensuality which are the consequences of defects in the early training of a child, that clogs our streets with prostitutes and our registers of births with entries of children born out of wedlock. In 1855, out of 85,532 births in London, 3455 were illegitimate ; and in 1857 there were known to the police, also in London, 8600 prostitutes, and 2825 houses of ill-fame. The number of illegitimate births, and number of known prostitutes form, however, but imperfect gauges of the amount of unchastity among unmarried females in the metropolis. In 1851, 3203 children were born out of wedlock in London, and in the same year the number of unmarried women was 212,293. If this proportion of unmarried women living unchastely to every illegitimate birth, be the same as that of married women to every legitimate birth, it would follow that at least 15,000 (about 1 in every 15) unmarried women living in London in that year were unchaste.

The protracted physical labour of the operative induces a condition of mind, apart from education or religious instruction, which disposes him to seek relaxation in sensuous rather than intellectual pleasures. When we consider, therefore, the as yet comparatively slight extent to which sound education has permeated the operative class, and the insufficiency of the means which exist for the literary accommodation and intellectual gratification of the class, it will follow as a natural consequence that such instruction as the majority of the class possess will be applied rather to the purpose of gratifying the passions than the intellect, and that the readiest and least troublesome method of indulging the senses and passions will be adopted. The gin-palace and the beer-house, the flaming decorations of the one and the cosey looks of the other, outshining the contracted, and perhaps crowded cottage, are flocked to, and probably comic songs, glees, or other attractions may be added to the seductive influences of gossip and strong drink. Many may keep within due bounds and never exceed either in one indulgence or another by frequently visiting these resorts; but when we have strained off the temperate, there is invariably found a dark and dense deposit of drunkenness and debauchery, fringed with want and wretchedness, recklessness and idleness, and capped with crime.

But the most important source of the degraded morality found among the operative class is the deficiency of religious and moral instruction. A great portion of this class is not only unprovided with the means of religious education, but it is inaccessible to the majority of the means already in existence. It is the deficiency of religious and moral instruction which makes the influence of the agencies of which we have spoken so injurious to the morality of the operative class. The effects of surrounding circumstances in depressing the standard of morality among a class of people, is in direct proportion to the deficiency in the religious and moral training of the class. The highest morality may coexist with the most unfavourable social conditions for the preservation of morality; but when there is a debasement of morality as a result of physical and social degradation, religious and moral instruction will prove of little benefit unless they are combined with measures for the promotion of physical and social improvement.

In 1848, an inquiry was made, by a committee of Fellows of the Statistical Society, into the condition of the labouring population of St. George's in the East, this parish being selected as affording an example of the average condition of the poorer classes of the metropolis. From the statistics obtained during this inquiry we may gather several illustrations of the remarks we have made upon the moral condition of the operative class of London. The total population of the district actually examined was 7711, of which number 3345 were children under 15 years of age. The number of houses occupied by this population was 1204, and the number of families inhabiting these houses, exclusive of single men and women lodgers, was 1802. Of these families, 636 occupied houses containing only one room, and 562 occupied houses containing two rooms. The number of individuals living in houses having only one room was 2025, that is 3·2 persons to each room; the number of individuals living in houses containing two rooms was 2·454, that is 2·2 persons to each room. These figures indicate a considerable degree of crowding, but they convey but a slight notion of the actual amount of crowding which occurs in sleeping apartments where a family of several children exist. In the houses containing two rooms, one room only is, as a general rule, used as a bed-chamber.

Evidence was sought as to the religious and moral character of the people by an inquiry into the form of religion professed by the heads of 1954 families, and by ascertaining the character of the literature principally used by them. The heads of 1328 families professed to belong to the Church of England, 64 to the Wesleyan Methodist Society, 177 to other denominations of Dissenters, 168 to the Roman Catholic Church, 35 were Jews, 150 professed no religion, 2 were Mahomedans, and of 30 the religion was not ascertained. "This extensive profession of attachment to the Gospel," remarks the Committee, "is a hopeful sign, though the limited extent to which the Wesleyan and other denominations of Dissenters appear to have penetrated into this mass of population, is rather remarkable, and will justify a feeling of doubt with regard to the profession made by some of belonging to the Established Church." This doubt is in a great measure confirmed by the evidence upon the means of spiritual instruction in the parish of St. George's in the East, given by the incumbent of the parish church and others before a Select Committee of the House of Lords last session. It would appear that the number of sittings already provided by the Church of England in St. George's in the East is 5,880, and that 18,019 additional sittings are required in order to provide for those who are not accommodated by any religious body. The parish church will seat about 1200 persons, but the incumbent states that it is not well attended. The average number of communicants at noon on Sunday is from 40 to 50, and the average number of attendants upon the week-day evening services is from 20 to 30, generally of the poorer classes; indeed, of those who attend the whole of the services, the larger proportion are, relatively, of the poor. The number of attendants at two rooms, which have been licensed for Divine service in the parish, would appear to be also small. At one service 30 individuals were counted in one of the rooms, and 48 were present at another time.

In 1260 families the Committee of the Statistical Society ascertained that the reading was confined almost wholly to newspapers, in 12 families only were other miscellaneous prints taken in, and in 29 only no newspaper whatever was taken. Upon these results the Committee remark, "This is not a cheering picture; the great use made of the capacity to read being, as far as this statement indicates, in ministering to mere excitement." The total number of books found in the district was 13,992, and these were distributed among 1954 families, 564 appearing to possess no books. Of the books, 58 were theatrical, 5,791 are classed as serious (including the Holy Scriptures and books of prayer), and 8153 as miscellaneous. It is not possible to ascertain statistically to what extent the "serious" books were read, but the impression of the agents of the committee was that, "in far the greater number of families which they visited, of all the books which they found in them, the Bible and Testament were those least read."

Of the children, 1266 attended infant, dame, and day schools, and 571 Sunday schools, and the payments of 1012 families for the schooling of their children did not exceed one shilling weekly.

In dealing with the moral pathology of the nether and operative classes of the metropolitan population, we have to do with results which are, in the main, the direct consequence of the social circumstances under which those classes are placed, and of a lack of true moral knowledge. But when we come to consider the indications of perverted morality which are found among the middle and aristocratic classes, it might be supposed that we should find that both crime and immorality had a more individualized and isolated character—that, indeed, crime and immorality would be seen rather as the result of individual depravity, than as a consequence of general social conditions inducing depravity. For the middle and higher classes do not lack, in appearance, either the means of both intellectual and moral knowledge, or the knowledge itself. When, however, we dip a little below the surface, we shall find that, as among the nether and operative classes, so among the middle and aristocratic classes, crime and immorality are mainly but the scum which floats upon the surface of loathsome pools of degraded and perverted social conditions. There are doubtless many and grave instances of individual depravity which have set at nought all training in uprightness, and that have broken through all the restraints of law, human and divine; but, as a rule, we may trace how acts of crime, and open immorality are ordinary consequences of the social conditions under which the individual has existed, and of a proclivity to vice due to defects in early training, or to evil influences acting in childhood and youth.

If we search into the method in which many trade transactions are ordinarily prosecuted, we at once obtain an important clue to the normal moral condition of many branches of the commercial class. In the evidence given on the Adulteration of Food and Drink before a Select Committee of the House of Commons in 1855, it was shown, with particular reference to London, that it was almost a general rule among the dealers in many of the most important articles of consumption to sell those articles in a more or less adulterated state, by which the value of the articles as food was diminished. Again, the formidable commercial disasters which have occurred during the last three or four years from fraudulent bill and banking transactions, and from malversations of funds, have shown that the lax principles of trading which led to such frightful disasters during the "railway mania," were no temporary principles, arising at the moment and disappearing with the occasion which seemingly gave rise to them, but that they were constantly present among the commercial class, and that the railway, bill-transaction, and banking disasters were epidemic manifestations of a low grade of trade morality.

It is not possible to state to what extent the degraded morality, of which the transactions mentioned are examples, prevails among the commercial class, but we know that men who have stood high in their particular class of business, and who have been esteemed of unblemished fame by the world,—men, indeed, who have been

> "Magnificently good,

> And held their heads up high like a giraffe,"

have fallen from practices which have resulted from this low grade of morality.

There is a common phrase in use among almost every section of commercial men, the "custom of the trade," which, upon examination, is often found to apply to a custom which has for its object the enhancing of the value of goods or property dealt in, by some market or business sleight, totally apart from the value of the goods or property. Several of these trade customs, as for example, the adulteration of articles of food, and certain lax proceedings of the share-markets, and in the use of accommodation bills, have become so rooted in many commercial transactions as to have become truly legitimate acts of trade, according to common usage. Nay, there is even an inner trade morality of these customs of the trade by which it is commonly understood that within certain limits only the trade practices referred to may be carried out. Now it is just in these little trade customs which have grown up from certain usages, and which are familiarly practised in many trades, that we have the germs of the majority of the huge frauds which arise out of inordinate speculation. In its ordinary form the little trade custom may be practised by every member of a trade alike, and with the majority of those who have been trained to this common morality of petty fraud, the usage which has taught them to practise it, has also sufficient power to keep them within the bounds which usage is pleased to dictate. But let inordinate desire for gain step in, let speculation become rife, let a temporary difficulty stand in the way which ordinary trade resources cannot remove, and the sleights legitimatized by the common custom of trade are apt to come forth in their true light. The barrier set up by usage cannot well withstand the pressure of necessity, and a "wicked whisper" is never wanting to suggest that if the fraudulent trade custom be right in principle in its less obtrusive form, it cannot be wrong in a more fully developed character; and the "honeyed hope of retrieving," (to use the words of John Sadleir's death confession) ever brightens the downward path with a delusive light.

The most remarkable study respecting petty fraud trade customs is the fact of their being practised frequently by men of religious habits. There is no doubt that there are many tradesmen who practise sleights of trade knowing them to be dishonest and with a dishonest intent; but there are also many tradesmen who practise sleights simply as trade customs in which they have been brought up, and who seem to be utterly ignorant, or incapable of appreciating their fraudulent character.

It would be painful to suppose that the wide-spread practice of adulteration of articles of food, which was shown to exist in the metropolis among all classes of dealers, by the Analytical Commission of the *Lancet*, was in every instance a deliberate act of dishonesty. The names and characters of many of the firms from whom articles were purchased for analysis would not justify such a supposition, unless we are prepared to adopt the conclusion of a perverted condition of morality existing among the middle class of London to which even that of the nether class is of a secondary character. The truth is, we believe, that these men have been taught from their earliest days to regard these practices as legitimate trade customs; they have grown up in this belief; it has never been tested by any other criterion than that of the trade; and the practice has become part and parcel of their normal mental and moral state, casting no dark shadow across their religious belief, and continuing until death, or until a popular outcry leads to an examination of the custom, or an Act of Parliament prohibits it, and so rouses, perchance, an individual to a true knowledge of the petty fraud he has been practising. It is to the existence of an insensibility to the fraudulent character of many trade customs, that we attribute those distressing scenes in which individuals who have brought upon themselves public obloquy by a fraudulent trade transaction, and even, perhaps, have been tried for it and convicted in a court of justice, have been retained in the full confidence of the religious body they have been connected with, and have received wide-spread sympathy from the branch of trade with which they have been connected, because they have acted simply in accordance with the "custom of the trade."

The habit of petty fraud which is but too commonly found to constitute the so-called "custom of the trade," forms frequently the particle of ferment which is ever ready to ferment actively under favourable circumstances, and to give rise to a more or less abundant crop of fraudulent commercial transactions.

But the low grade of morality of which certain trade customs are an indication is not manifested in those customs alone. They are the natural result of a degraded morality, but they are most commonly met with side by side with careless and indifferent feelings towards the requirements of strict morality generally. This indifference is indeed the main form in which a low grade of morality is found among the middle and higher classes of society. Conventional morality, that is, propriety both in speech and manners, is now almost an essential requisite of every section of the middle and higher classes of society, and formal religion has become fashionable; yet, with all this improvement, a vast amount of immorality still nestles within the skirts of the middle and higher classes, and is tolerated by them—nay, it would appear to be expected by a considerable portion of these classes that youth should go through a certain portion of immorality, as a matter of course, before it comes to manhood, the so-called "sowing of wild oats" being, in fact, regarded as a normal phase of humanity.

The laxity of morals which exists among the youth of the higher and middle classes of society is partly the result of the indifference with which it is regarded by a large section of society, and partly a consequence of the education given to the youth. The education is directed more to the cultivation of the intellectual than of the moral faculties. Such training as is given to the moral faculties, when these are not altogether, as is often the case, left to be formed simply by example at home or at the schools, is almost invariably of a most imperfect character. It consists usually in the teaching of the bare precepts of morality, and it is rarely accompanied by any satisfactory training in the application of those precepts. If the moral faculties, so far as developed, have not already been perverted, as is not unfrequently the case, before the youth has left school and commenced to rough it in the world, he enters into the struggle of life almost altogether ignorant of the peculiar dangers he is exposed to, and consequently liable to become a prey to the first temptation that falls in the way.*

* ". . . . 'She (my mother) brought me up to pray and hope that I might some day be converted, and become a child of God. . . . And one could not help wishing to enjoy oneself as much as possible before that event happened.' 'Before that event happened, my dear fellow? Pardon me, your tone is somewhat irreverent.' 'Very likely. I had no reason put before me for regarding such a change as anything but an unpleasant doom, which would cut me off, or ought to do so, from any field sports, from poetry, from art, from science, from

Abstract principles of morality, of which the significance and application are imperfectly apprehended, offer but slight obstacles to the evil effects of sensual pleasures. Moreover, the practice of morality being left to be formed chiefly after entrance into active life, the youth is apt to take example from that form of practice which is least exacting. A morality of this species sees no wrong in those gambling transactions, so fruitful of ruin, depravity, and crime, which constitute a marked characteristic of many of the amusements of the higher classes, and particularly of those connected with the so-called "sporting world," and it justifies a special code of morality for the guidance of such transactions, this code being the analogue of the "custom of the trade" among the commercial class. But a main character of this low grade of morality (which might with truth be termed secular morality) is, that it nourishes an appetite for pleasure, using that word in the conventional acceptation of the term as applied to popular amusements. Secular morality, however, offers but a feeble resistance —nay, it too frequently co-exists with a tendency to succumb to the more pernicious effects of pleasure. A striking example of the insidious and dangerous character of this degraded morality is witnessed at the present time, among the highest classes of society, in the patronage and support which has been given to the opera of "La Traviata," the "Street-walker." In this opera is represented an episode in the life of a common prostitute, in which an overweening attachment, less noble in character than that of a dog to its master, is upheld to admiration and sympathy; and Christianity is parodied in a fashion little short of being blasphemous, by this attachment being exhibited as a sufficient justification for sin and preparation for heaven!

The commoner effects of the low grade of morality existing among the middle and higher classes may be seen in their most politics—for Christians, I was told, had nothing to do with the politics of the world,—from man and all man's civilization in short, and leave to me, as the only two lawful indulgences, those of living in a good house and begetting a family of children.' 'And did you throw off the old creeds for the sake of the civilization which you fancied they forbid?' 'No. . . . . I am a Churchman, you know, principally on political grounds, or from custom, or from —the devil knows what, perhaps—I do not.' 'Probably it is God and not the devil, who knows why, Templeton?' 'Be it so. . . . . Frightful as it is to have to say it. . . . . I do not much care. . . . . I suppose it's all right; if it is not, it will come right at last. And, in the meantime, I compromise, like the rest of the world. . . . . And so, I believe, I am going to have no religion at all, and no substitute for it either. . . . . I am becoming more and more of an animal,—fragmentary, inconsistent, seeing to the root of nothing, unable to unite things in my own mind. I just do the duty which lies nearest and looks simplest. I try to make the boys good, plucky, and knowing—though what's the use of it? They will go to college with even less principles than I had, and will get into proportionately worse scrapes. I expect to be ruined by their debts before I die.'"—*Phaeton; or, Loose Thoughts for Loose Thinkers*, by C. Kingsley, pp. 75—80.

conspicuous characters in the casinos and principal haunts of vice in the metropolis. These grand portals of iniquity are crowded nightly with our youth. Here they graduate in sensuality and vice; and to the crash of music, and the quick paces of the dance, they are whirled rapidly into a vortex of sin, in the inner whirl of which is found a depravity which, sooner or later, leads to ruin. But few may pass even into the outermost whirls of the vortex and thereafter escape fully from the taint of dark vice. The moral faculties are still further blunted by the briefest acquaintance with these haunts, and the lessons of sensuality learnt in them are carried into domestic life, and the terrible results of these lessons are from time to time made known in the records of the Divorce, Criminal, and other Courts of Justice.

The great number of splendid resorts for the mere gratification of sensual desires which exist in London, are at once an indication of the extent to which a low grade of morality exists among the middle and higher classes, and a cause of the maintenance of that low grade. These resorts are not the less dangerous because they are conducted, in the main, with due regard to external propriety. The splendid drinking-saloon, with its attractions of song and music, most surely induces the insatiate craving for strong drink, with the necessary concomitants of drunkenness and allied vices; and the dancing-saloon most certainly paves the way to the innermost recesses of iniquity, though the externalisms of decency may never be departed from within it. Nay, the very aspect of grace which is thrown over these resorts adds but to the subtlety of the evil:—

"The devil's most devilish when respectable."

Sensual gratifications and pleasures are at the best but treacherous indulgences; and the man who has been most carefully trained in the right may hardly at times hold his own against them. But when these pleasures are heightened by careful development, when they are divested of every moral check beyond the ordinary requirements of secular morality, and when their object is merely to minister to the gratification of the moment, then they may become most pernicious.

The low grade of morality which pervades a large portion of the middle and higher classes, is not shown alone in an undue proclivity to sensual gratifications, and in the fostering of dubious special codes of morality, of which the extreme results are crime and overt immorality. These are its most prominent effects; but its influence is also widely and most injuriously felt in an active tendency to depress the standard of religious practice, and in a lowering effect upon the morality of the operative and nether class, by degrading the bonds of relationship between the master and the servant, between the workman and his employer, and between the landlord and tenant—these bonds being too commonly regarded as having no higher character than personal service on the one hand, and pounds, shillings, and pence on the other.

Of the different results which arise from the low grade of morality that prevails, to a greater or less extent, among all classes of the metropolitan population, one only—*crime*—can be estimated numerically. The amount and character of the crime which occurs in London from year to year, as well as the number of persons arrested, convicted, and punished, are recorded in the returns of the City of London and of the Metropolitan Police (the police of the metropolis consisting of two principal divisions, the duties of one of which are confined to the comparatively contracted area of the City of London proper, while the duties of the other extend to the remainder of the metropolis), and in the "Judicial Statistics" which are compiled by order of the Secretary of State for the Home Department.* From the latter source it would appear that, in 1857, 8452 indictable offences were committed in London, and that for these offences 6618 persons were apprehended, of whom 2973 were discharged, 3 were bailed, 73 were bailed for trial, 24 were committed for want of sureties, and 3545 were committed for trial. In the same year there were arrested, also in London, for offences with which the magistrates dealt summarily, 103,671 persons (71,975 males, 31,696 females), of whom 47,620 were discharged, and 56,051 convicted. The whole number of arrests for indictable offences, and for offences with which the magistrates dealt summarily, was, during the year, 110,289, which would give an average of somewhat more than 302 arrests daily. The character of the persons arrested and proceeded against, as far as could be ascertained, was as follows:—

| —— | Known thieves. | Prostitutes. | Vagrants and tramps. | Suspicious characters. | No known occupation. | Previous good character. | Character unknown. |
|---|---|---|---|---|---|---|---|
| Indictable offences . | 749 | 393 | 56 | 2,070 | 82 | 1,215 | 2,053 |
| Offences summarily disposed of | 9,898 | 5198 | 4107 | 11,547 | 1488 | 24,582 | 46,851 |
| | 10,647 | 5591 | 4163 | 13,617 | 1570 | 25,797 | 48,904 |

* The writer of the article "On the Moral Pathology of London" would here express his thanks to Sir Richard Mayne, the Chief Commissioner of Metropolitan Police, and to Samuel Redgrave, Esq., the Criminal Registrar at the Home Office, for their kindness in furnishing him with the criminal statistics made use of in the article. To Mr. Redgrave belongs the honour of having drawn up, and, we believe, also, suggested the series of returns published by Government under the title of

Thus 1 in every 10·4 of the individuals arrested belonged to the criminal class; 1 in every 4·04 was of bad or indifferent character, and 1 in every 4·07 was of previously good character. Nearly two-thirds of the whole number of persons arrested were formerly individuals of previously good character and of whom the character is unknown; and doubtless the greatest portion of these are to be regarded as having for the first time committed offences, or been suspected of having committed offences against the law. The statistics of character are not applied to persons actually convicted of crime, consequently we cannot estimate with any nearer degree of correctness than that given in the foregoing table, the number of new criminals added to the police-lists during the year; but the data are sufficient to show the great preponderance of persons who for the first time had brought themselves within the grasp of the law. Perhaps the most remarkable item in the table is the number of prostitutes arrested. The number of prostitutes known to the police in 1857 amounted, as has been previously stated, to 8600. It follows, therefore, that more than two-thirds of the whole number were under arrest during that year.

The "Judicial Statistics" for 1857 contain, for the first time, the Criminal Returns of the London Police, consequently these cannot be compared with any previous statistics of a similar character. We have at our command the Criminal Returns of the Metropolitan division of Police only, and the following data are derived from this source. As these details apply only to a portion, although by far the greatest portion of the metropolis, it must be borne in mind that they show simply, approximatively, the variation in amount and character of the crime of London.

In 1857 the Metropolitan Police took 79,364 persons into custody, of whom 37,564 were discharged by the magistrates, 38,731 were summarily convicted or held to bail, and 3069 were committed for trial, of whom 2460 were convicted and sentenced, 502 were acquitted, and 107 were not prosecuted, or bills were not found against them. During the five years, 1852–56, the annual average of arrests was 72,586; of persons discharged by the magistrates 37,361; of charges summarily disposed of, or held to bail, 31,014; of committals for trial, 4210; of convictions, 3467; of acquittals, 624; and of charges not prosecuted, or bills not found, 117. The gross amount of arrests and of cases summarily disposed of and committed for trial in 1857 is, therefore, considerably in excess of the gross annual average of arrests and of charges summarily disposed of and committed for trial during the preceding five years; and although a decrease appears in the amount of committals in 1857, this is

"Judicial Statistics." In the recently published volume of these statistics the criminal returns only for England and Wales are given, but in subsequent volumes it is intended to publish returns also of the proceedings in the Common Law, Equity, Canon, and Civil Law Courts. When the whole of the returns projected by Mr. Redgrave are completed and regularly filled up, the statistics of justice in this kingdom will probably be more complete than those of any other kingdom.

probably due to the larger number of cases disposed of summarily by the magistrates. Does the increase in the amount of crime during 1857 above the average of the preceding five years indicate an actual increase in the quantity of crime in London, or an increase proportionate only to the increase of population during that and the previous years? Estimating the population roughly by adding the excess of births each year to the population of the previous years, commencing with the ascertained population of 1851, we arrive at the following results:—

*Population of London, according to the Census of* 1851, 2,362,236.

| — | Excess of births. | Population. | Number of arrests. | Proportion to population. | Number of summary convictions & committals. | Proportion to population. |
|---|---|---|---|---|---|---|
| 1852 . . | 26,612 | 2,388,848 | 73,257 | 1 in 32·06 | 34,985 | 1 in 68·02 |
| 1853 . . | 22,185 | 2,411,033 | 72,316 | 1 in 33·03 | 34,695 | 1 in 69·04 |
| 1854 . . | 11,188 | 2,422,221 | 75,614 | 1 in 32·00 | 35,245 | 1 in 68·07 |
| 1855 . . | 23,590 | 2,445,811 | 68,505 | 1 in 35·07 | 33,655 | 1 in 72·03 |
| 1856 . . | 20,893* | 2,466,704 | 73,240 | 1 in 33·06 | 36,689 | 1 in 67·02 |
| 1857 . . | 20,893* | 2,487,597 | 79,364 | 1 in 31·03 | 41,800 | 1 in 59·09 |

* Average of the four years, 1852–55.

If these calculations be received as an approximation to the proportion of arrests, summary convictions, and committals, to the total population for the years 1852–57, it will follow that there was a considerable excess in the amount of arrests, summary convictions, and committals during 1857. It would also appear that in 1855 there was a considerable diminution as well in the number of arrests, as of the summary convictions and committals.

The offences which come under the cognizance of the police are divided in the official returns into six categories. In the first category are enumerated "Offences against the person;" to wit, murder, attempts to murder, manslaughter, rape, bestiality, and other libidinous crimes, extorting money under threats of violence, common assaults and assaults on the police, attempting to commit suicide, &c. The second category contains "Offences against property committed with violence;" for example, burglary, breaking into dwelling-houses, shops, &c., robbery, assaults with intent to rob, &c. The third category contains "Offences against property committed without violence;" for example, larceny, cattle, sheep, and horse stealing, embezzlement, receiving stolen goods, unlawful possession of goods, &c. The fourth category contains "Malicious offences against property;" to wit, arson and wilful damage. The fifth category contains "Forgery, and offences against the currency." The sixth category contains "Other offences not included in the foregoing categories;" for example, drunkenness, drunk and disorderly characters, vagrancy, disorderly characters, offences under the Metropolitan Police Act, the Hackney Carriage Act, the Juvenile Offenders Act, &c.

The number of persons who were (*a*) summarily convicted or held to bail, or (*b*) committed for trial, in each of the different categories in 1857 was as follows: I. *Offences against the person* (*a* 7750, *b* 284) 8034; II. *Offences against property committed with violence* (*b*) 293; III. *Offences against property committed without violence* (*a* 9417, *b* 2143) 11,550; IV. *Malicious offences against property* (*a* 1941, *b* 15) 1956; V. *Forgery and offences against the currency* (*b*) 292; VI. *Other offences not included in the above classes* (*a* 18,403, *b* 32) 18,435. The annual average of the number of persons summarily convicted and held to bail, or committed for trial in the different classes of offences, during the five years, 1852 to 1856, was as follows: Class I. (*a* 6146, *b* 267), 6683; Class II. (*b*) 255; Class III. (*a* 6046, *b* 3409), 9455;

Class IV. (*a* 1448, *b* 7), 1495; Class V. (*b*) 306; Class VI. (*a* 17,462, *b* 25), 17,487. In each class of offences, with the exception of the fifth class (Forgery and offences against the currency), there was an excess in 1857 above the mean of the previous five years.

The total number of persons who were upon trial, convicted and sentenced in 1857 was 2460; the mean annual number of convictions upon trial during the five years, 1852–56 being 3467. On the other hand, however, the number of offenders summarily dealt with, or held to bail, by the magistrates in 1857, was 38,731, while the annual mean of the preceding five years was 31,014; showing, as has been previously intimated, that the diminution of the amount of convictions after trial was due to a larger number of charges being dealt with summarily by the magistrates.

The crimes which seem to be most influential in determining the increment or decrement of the amount of crime, are those which are dealt with summarily, as may be gathered from the following statement of the number of individuals summarily convicted or held to bail, and committed for trial during the five years, 1853–57:—

| — | Summarily convicted or held to bail. | Committed for trial. | Convicted and sentenced. |
|---|---|---|---|
| 1853 . . . . | 30,327 | 4368 | 3613 |
| 1854 . . . . | 30,941 | 5159 | 4304 |
| 1855 . . . . | 29,796 | 3859 | 3169 |
| 1856 . . . . | 33,451 | 3238 | 2587 |
| 1857 . . . . | 38,731 | 3069 | 2460 |

The annual average of summary convictions during the five years is 32,649, and the greatest excess above the average (in 1857) was 6082; but the difference between the lowest number of convictions in the period and the highest is no less than 8935, the variation in the number of committals being 2090, while the annual average of committals was 3938.

The offences for which the greatest number of persons were convicted in the six years, 1852–57, are set forth in the following table:—

| — | 1852. | 1853. | 1854. | 1855. | 1856. | Mean of the five years. | 1857. |
|---|---|---|---|---|---|---|---|
| Drunkenness and drunk and disorderly characters . . . . . . . | 8366 | 8240 | 7273 | 6376 | 6305 | 7312 | 7759 |
| Disorderly characters and disorderly prostitutes. | 3949 | 3955 | 3630 | 3562 | 4008 | 3820 | 4889 |
| Vagrancy . . . . . | 2168 | 1860 | 2065 | 1628 | 1956 | 1936 | 2485 |
| Suspicious characters and reputed thieves . | 869 | 830 | 798 | 871 | 1025 | 678 | 1099 |
| Common assaults and assaults on the police . | 6436 | 6188 | 6280 | 6921 | 6138 | 6392 | 7505 |
| Larceny . . . . . | 2491 | 3170 | 3772 | 2755 | 1907 | 2819 | 1765 |
| Unlawful possession of goods. . . . . . | 3694 | 4317 | 5765 | 5019 | 8160 | 5551 | 8817 |
| Wilful damage . . . | 1357 | 1382 | 1435 | 1504 | 1742 | 1484 | 1941 |

The greatest number of convictions for particular offences occur for drunkenness, drunk and disorderly conduct, common assaults, assaults on the police, the unlawful possession of goods, and disorderliness.

The drunk, and drunk and disorderly characters hold the most prominent position in the list of offenders,* but the figures in the foregoing table give but an imperfect notion of the extent to which the vice of drunkenness prevails openly. The following table shows the number of arrests and convictions for drunkenness, and drunk and disorderly conduct during the ten years, 1848–57, as well as certain incidental data which will aid in the formation of a better comprehension of a few of the evils which arise from habits of indulging to excess in spirituous liquors:—

| — | Drunkenness & drunk & disorderly conduct. | | | | Illicit distillation. |
|---|---|---|---|---|---|
| | Arrests. | Summary convictions. | Males. | Females. | |
| 1848 . . . . | 16,461 | 4956 | 3185 | 1771 | 46 |
| 1849 . . . . | 22,027 | 6849 | 4457 | 2392 | 89 |
| 1850 . . . . | 23,897 | 8342 | 5586 | 2756 | 100 |
| 1851 . . . . | 23,172 | 8027 | 5129 | 3898 | 100 |
| 1852 . . . . | 23,640 | 8366 | 5526 | 2840 | 90 |
| 1853 . . . . | 23,652 | 8240 | 5464 | 2776 | 50 |
| 1854 . . . . | 22,078 | 7273 | 4806 | 2472 | 56 |
| 1855 . . . . | 19,297 | 6376 | 4025 | 2351 | 65 |
| 1856 . . . . | 18,703 | 6305 | 3796 | 2509 | 27 |
| 1857 . . . . | 20,047 | 7759 | 4790 | 2969 | 18 |

| — | Number of public-houses and beer-shops summoned by the police, and the number convicted and dismissed. | | | Deaths from intemperance. | Deaths from delirium tremens. |
|---|---|---|---|---|---|
| | Convicted. | Dismissed. | Total. | | |
| 1848 . . . . | 762 | 158 | 920 | 57 | 141 |
| 1849 . . . . | 1125 | 247 | 1372 | 62 | 164 |
| 1850 . . . . | 1085 | 269 | 1354 | 74 | 157 |
| 1851 . . . . | 960 | 226 | 1186 | 64 | 136 |
| 1852 . . . . | 1293 | 321 | 1614 | 73 | 148 |
| 1853 . . . . | 1138 | 263 | 1401 | 73 | 152 |
| 1854 . . . . | 1067 | 290 | 1357 | 84 | 174 |
| 1855 . . . . | 718 | 256 | 974 | 83 | 164 |
| 1856 . . . . | 881 | 229 | 1110 | — | — |
| 1857 . . . . | 917 | 235 | 1152 | — | — |

The following table shows the number of convictions during the ten years, 1848–57, for several of the most serious offences against the laws:—

* The offence for which the greatest number of persons were arrested, in the whole of England and Wales, during 1857, was *assault*. It must not, however, be inferred, therefore, that there is an excess of drunkenness and drunk and disorderly characters in London as compared with other districts, or with the whole of the kingdom, for the arrests for drunkenness, and drunk and disorderly characters will depend upon the activity and efficiency of the police, and will be greater in proportion in cities and large towns than in country districts. It is not improbable that when the arrangements for the rural police have been fully carried out, we shall find an increase in the number of arrests for drunkenness, and drunk and disorderly characters, in the criminal returns for the whole kingdom.

| — | Murder. | | Attempts to murder. | | Manslaughter. | | Rape, and assaults with intent to commit rape. | |
|---|---|---|---|---|---|---|---|---|
| | Com. | Conv. | Com. | Conv. | Com. | Conv. | Com. | Conv. |
| 1848 . . . . . | 13 | 5 | 78 | 60 | 14 | 3 | 37 | 29 |
| 1849 . . . . . | 17 | 7 | 80 | 65 | 21 | 11 | 27 | 17 |
| 1850 . . . . . | 6 | 4 | 73 | 56 | 18 | 9 | 32 | 18 |
| 1851 . . . . . | 13 | 9 | 74 | 55 | 21 | 13 | 49 | 32 |
| 1852 . . . . . | 11 | 6 | 72 | 60 | 15 | 6 | 45 | 29 |
| 1853 . . . . . | 7 | 2 | 64 | 47 | 10 | 5 | 21 | 16 |
| 1854 . . . . . | 10 | 5 | 46 | 36 | 13 | 6 | 28 | 12 |
| 1855 . . . . . | 12 | 6 | 60 | 56 | 18 | 6 | 15 | 9 |
| 1856 . . . . . | 11 | 6 | 62 | 50 | 8 | 4 | 19 | 11 |
| 1857 . . . . . | 13 | 9 | 71 | 62 | 13 | 5 | 31 | 18 |

| — | Burglary, and breaking into dwelling-houses, &c. | | Robbery. | | Forgery and offences against the currency. | |
|---|---|---|---|---|---|---|
| | Com. | Conv. | Com. | Conv. | Com. | Conv. |
| 1848 . . . . . | 188 | 154 | 100 | 82 | 208 | 190 |
| 1849 . . . . . | 188 | 156 | 48 | 37 | 447 | 120 |
| 1850 . . . . . | 195 | 162 | 65 | 44 | 154 | 136 |
| 1851 . . . . . | 162 | 131 | 79 | 54 | 290 | 190 |
| 1852 . . . . . | 187 | 149 | 46 | 35 | 315 | 282 |
| 1853 . . . . . | 166 | 138 | 42 | 33 | 300 | 277 |
| 1854 . . . . . | 196 | 168 | 54 | 39 | 377 | 366 |
| 1855 . . . . . | 151 | 132 | 56 | 42 | 251 | 216 |
| 1856 . . . . . | 276 | 239 | 98 | 76 | 289 | 237 |
| 1857 . . . . . | 218 | 192 | 73 | 55 | 302 | 246 |

If we divide this table into two quinquennial periods, and ascertain the mean number of convictions for the different offences enumerated in each of the periods, we shall find that, during the five years, 1853–57, the mean number of convictions for all the offences, except burglary and breaking into dwelling-houses, &c., and forgery and offences against the currency, is less than in the five years, 1848–52 :—

| | Murder. | Attempts to murder. | Manslaughter. | Rape, &c. | Burglary, &c. | Robbery. | Forgery, &c. |
|---|---|---|---|---|---|---|---|
| | Mean. | Mean. | Mean. | Mean. | Mean. | Mean. | Mean. |
| 1848–52 . . . | 6·3 ... | 59·1 ... | 8·2 ... | 25· ... | 150·2 ... | 50·2 ... | 183·3 |
| 1853–57 . . . | 5·3 ... | 50·1 ... | 5·1 ... | 13·1 ... | 173·4 ... | 49· ... | 222·1 |

If these figures might be taken as indices by which to measure the actual quantity of offences committed, they would show a gratifying decrease in the amount of serious crime in the metropolis. In the absence, however, of data showing the actual number of grave offences which have occurred, conclusions derived from the number of convictions, as to the amount of crime, must be received with caution.

The ages, sex, and degree of instruction of the persons convicted during the five years, 1853–57, are set forth in the following tables :—

AGE.

| | Under 10 years. | 10 to 15. | 15 to 20. | 20 to 25. | 25 to 30. |
|---|---|---|---|---|---|
| 1853-57 . . . | 518 | 12,299 | 34,480 | 39,349 | 26,308 |

| | 30 to 40. | 40 to 50. | 50 to 60. | 60 and upwards. |
|---|---|---|---|---|
| 1853-57 . . . | 36,179 | 19,086 | 7891 | 3409 |

SEX.

| | Under 10 years. | | 10 to 15. | | 15 to 20. | | 20 to 25. | | 25 to 30. | |
|---|---|---|---|---|---|---|---|---|---|---|
| | M. | F. | M. | F. | M. | F. | M. | F. | M. | F. |
| 1853-57 | 465 | 53 | 11,158 | 1141 | 25,985 | 8495 | 27,389 | 11,960 | 16,621 | 966 |

| | 30 to 40. | | 40 to 50. | | 50 to 60. | | 60 and upwards. | |
|---|---|---|---|---|---|---|---|---|
| | M. | F. | M. | F. | M. | F. | M. | F. |
| 1853-57 | 22,304 | 13,075 | 11,686 | 7400 | 5078 | 2840 | 2215 | 1194 |

INSTRUCTION.

| | Total. | | Neither read nor write. | | Read only, or read and write imperfectly. | | Read and write well. | | Superior instruction. | |
|---|---|---|---|---|---|---|---|---|---|---|
| | M. | F. | M. | F. | M. | F. | M. | F. | M. | F. |
| 1853-57... | 123,781 | 55,598 | 21,410 | 13,230 | 91,823 | 40,997 | 9424 | 1327 | 1124 | 44 |

The ages of 20 to 25 and 30 to 40 include the greatest number of persons convicted; and, of the whole amount of criminals, the largest number were under 30 years of age. The increase and decrease of crime in a district is due principally to the criminality of individuals of mature years—of those, in fact, who have arrived at an age when they become most fully exposed to the vicissitudes of life. The proportion of female criminals is large, being somewhat more than one-third of the whole amount. The bulk of the criminals consisted of persons who had received an imperfect education. This is the rule among the criminal class. A vast amount of instruction of a most unsatisfactory character, and in which the moral faculties, in particular, are neglected, prevails among the lower classes and also among the middle class; and it is from individuals who have received this spurious education that the criminal class chiefly derives its additions.

The statistics of felonies against property constitute a very interesting portion of the Police Returns. In the following table is given a summary of the number of felonies which occurred during the five years, 1853–57, the number of convictions for felony during the same period, the first amount of loss, the amount recovered, by the police, and the total amount of loss:—

| —— | Number of felonies. | Number of persons tried and convicted. | Amount of first loss. | Amount recovered. | Amount of total loss. |
|---|---|---|---|---|---|
| 1853 . . . . | 11,699 | 2728 | £41,988 | £12,458 | £29,530 |
| 1854 . . . . | 13,765 | 3341 | 49,898 | 9,780 | 40,069 |
| 1855 . . . . | 14,175 | 2535 | 49,996 | 10,216 | 39,780 |
| 1856 . . . . | 15,621 | 2029 | 53,193 | 11,363 | 41,830 |
| 1857 . . . . | 16,551 | 1808 | 51,832 | 10,577 | 41,255 |

The particular felonies of which summaries are given in the foregoing table form a curious list. The following table shows the number and character of the felonies committed in the Metropolitan Police district during the year 1857:—

| Description of Felony. | | | Number. | Number of persons tried and convicted. | Amount of loss. First loss. | Amount of loss. Amount recovered. | Amount of loss. Total loss. |
|---|---|---|---|---|---|---|---|
| | | | | | £ | £ | £ |
| | | Burglary ... ... ... ... ... | 129 | 127 | 2004 | 618 | 1386 |
| | | With violence to persons ... | — | 2 | — | — | — |
| | | By lifting windows or entering doors not fastened ... | 86 | 26 | 1042 | 56 | 986 |
| | | Breaking into a dwelling-house, &c. ... ... ... ... | 40 | 39 | 490 | 134 | 356 |
| | | Building, shop, &c. ... ... | 107 | 21 | 685 | 112 | 573 |
| | | *Embezzlement ... ... ... | 209 | 101 | 1531 | 192 | 1339 |
| | | *Forgery ... ... ... ... ... | 20 | 36 | 263 | 14 | 249 |
| | | *Fraud ... ... ... ... ... | 522 | 92 | 2783 | 319 | 2464 |
| | | Robbery on highway ... ... | 21 | 44 | 47 | 12 | 35 |
| | | Horse stealing ... ... ... ... | 24 | 16 | 488 | 401 | 87 |
| | | Cattle stealing ... ... ... ... | 9 | 5 | 90 | 74 | 16 |
| | | Sheep stealing ... ... ... ... | 30 | 8 | 84 | 44 | 40 |
| | | Dog stealing ... ... ... ... | 17 | — | 99 | 28 | 71 |
| Larcenies. | Common | Goods, &c. exposed for sale... | 2300 | 238 | 1908 | 760 | 1148 |
| | | Tools, lead, glass, &c. from unfinished houses ... ... | 975 | 72 | 768 | 168 | 600 |
| | | From carts or carriages ... | 351 | 24 | 833 | 230 | 603 |
| | | Linen, &c. exposed to dry ... | 350 | 20 | 255 | 32 | 223 |
| | | Poultry, &c. exposed in an outhouse ... ... ... ... | 817 | 64 | 735 | 167 | 568 |
| | In a dwelling-house, &c. | By false keys only ... ... | 229 | 20 | 1753 | 65 | 1688 |
| | | *By lodgers ... ... ... ... | 1550 | 74 | 4019 | 768 | 3251 |
| | | *By servants ... ... ... ... | 1781 | 140 | 10,385 | 3017 | 7368 |
| | | By doors being left open ... | 2570 | 209 | 5890 | 985 | 4905 |
| | | *By false messages, &c. ... | 632 | 29 | 1835 | 184 | 1651 |
| | | By lifting up windows or breaking glass ... ... ... | 611 | 30 | 2603 | 196 | 2407 |
| | | By attic windows, through empty houses ... ... ... | 30 | — | 900 | — | 900 |
| | | By means unknown ... ... | 119 | 8 | 336 | 15 | 321 |
| | From the person | Picking pockets ... ... ... | 1608 | 249 | 5229 | 1138 | 4091 |
| | | *From drunken persons ... | 146 | 10 | 688 | 83 | 605 |
| | | From children ... ... ... ... | 106 | 2 | 59 | 4 | 55 |
| | | *By prostitutes ... ... ... | 854 | 79 | 3542 | 564 | 2978 |
| | | Larcenies on the River Thames | 228 | 23 | 488 | 197 | 291 |
| | | Total ... ... ... | 16,551 | 1808 | 51,832 | 10,577 | 41,255 |

It is evident that the cases marked thus (*) could not have been prevented by the police.

The number of convictions for felony during the years 1853–57 was 12,441; the ascertained loss of property from felony, in the same period, amounted to 192,464*l*. If we suppose that the whole of this sum (which would not be the case, as stolen property, except when money, can only be got rid of for sums much beneath its value) was divided solely among the number of individuals convicted for felony, 15*l*. and a few odd shillings only would fall to the lot of each person! Crime is, indeed, a most unprofitable occupation.

Of the 12,441 individuals committed for felony during the quinquennial period, 1853–57, 3180 had been in custody for felony twice previously, 689 thrice, 147 four times, and 53 five times and upwards.

The great increase in the total amount of offenders, as well as in the number of arrests and convictions for several of the slighter offences, which is shown by the preceding statistics to have occurred in 1857, and the decrease of arrests and convictions in 1855, are not susceptible of explanation by the information which we at present possess. Perhaps the most important conclusion which may be derived from the criminal statistics of the metropolis is, that the greatest number of convictions and arrests occur for offences which arise from a low grade or a perverted state of morality, rather than from more deliberate criminality. This conclusion affords additional support to the propositions which we have endeavoured to set forth in this article.

In addition to the returns of the number of persons taken into custody, the offences for which they have been arrested, and the results of the arrests, the police also make certain "Miscellaneous returns," among which is included a report of the "Number of suicides committed, and the number attempted and prevented by the police and others."

The numerical records of suicide form a terrible illustration of the acuteness of the misery and sin which exists in the metropolis. The statistics of suicide are doubtless incomplete, as it is not improbable that many of those deaths, upon an inquiry into the cause of which a jury returned a verdict of "*Found dead,*" have resulted from suicide. The number of instances in which a verdict of 'Found dead' was returned, in the county of Middlesex last year, was 535. The following table shows the number of suicides and attempted suicides in the Metropolitan Police District during the ten years 1848–57:—

| — | Number of suicides committed, and the number attempted and prevented by the police. | | |
|---|---|---|---|
| | Suicides committed. | Suicides attempted and prevented by the police. | Suicides attempted and otherwise prevented. |
| 1848 . . . . . | 100 | 11 | 67 |
| 1849 . . . . . | 131 | 15 | 60 |
| 1850 . . . . . | 140 | 10 | 78 |
| 1851 . . . . . | 120 | 8 | 67 |
| 1852 . . . . . | 109 | 7 | 74 |
| 1853 . . . . . | 131 | 7 | 110 |
| 1854 . . . . . | 118 | 14 | 72 |
| 1855 . . . . . | 116 | 13 | 60 |
| 1856 . . . . . | 127 | 12 | 66 |
| 1857 . . . . . | 154 | 6 | 87 |

We may add, as a pendant to this melancholy table, that in the eight years 1848–55, 242 persons died in the metropolis from *privation* of food, 49 from *neglect*, and 109 from *cold*.

In the preceding article our object has been to show the chief points connected with the etiology of a low grade of morality which we believe to exist extensively among all classes of the population of London (and, indeed, of the kingdom), and which we consider to be the substratum of crime and other forms of immorality. We have attempted to indicate the most prominent causes which, acting upon this substratum, determine overt acts, not only of crime, but of vice generally; and we have given a summary of the crime which has occurred in London during the last five years, as an illustration of one of the effects of the low grade of morality existing among the metropolitan population.

